# Phenotypic & genetic characterization of *Bacillus cereus* isolated from the acute diarrhoeal patients

**Published:** 2011-01

**Authors:** Mousumi Banerjee, Gopinath B. Nair, Thandavarayan Ramamurthy

**Affiliations:** *Department of Bacteriology, National Institute of Cholera & Enteric Diseases (ICMR), Kolkata, India*

**Keywords:** *B. cereus*, BCET, diarrhoea, PFGE, virulence genes

## Abstract

**Background & objectives:**

*Bacillus cereus* is one of the pathogens responsible for human diarrhoea, mainly due to consumption of contaminated food. The present study was undertaken to determine the occurrence of *B. cereus* among diarrhoeal patients and its phenotypic and genetic characteristics that determine the virulence and clonal features.

**Methods:**

Stool specimens were collected for two years from acute diarrhoeal patients attending the two referral hospitals in Kolkata. Presence of virulence genes in *B. cereus* was determined by PCR. Clonality was assessed by pulsed-field gel analysis (PFGE) by restriction digestion with *Sma*I and *Not*I enzymes. Enterotoxins were detected by haemolysin assay and using BCET-RPLA kit. Invasion assay was done on Hep-2 cell line. Antimicrobial susceptibility was tested by disc diffusion method.

**Results:**

*B. cereus* was identified in 54 (3.5%) of the 1536 diarrhoeal cases studied. Majority of the isolates were susceptible to many antibiotics but showed resistant to amoxyclav and cephalosporins. Six genes covering the two different enterotoxic complexes determining the pathogenicity of *B. cereus* have been characterized by PCR. The *nhe* genes were detected in a higher proportion than *hbl*. Except in two, clonal diversity was noticed among 21 *B. cereus* isolates. Haemolytic enterotoxin was detected in 76 per cent of the isolates. Majority of the isolates (67%) produced *in vitro* enterotoxin (BCET) confirming its involvement in the infection.

**Interpretation & conclusions:**

Though the presence of *B. cereus* was not high in patients with diarrhoea, several virulence factors confirm their association with diarrhoea. Distinct clonality was identified in majority of the isolates indicating their origin from different sources.

Acute diarrhoea is an endemic disease in many parts of India especially, in Gangetic Bengal. *Bacillus cereus* is one of the pathogens responsible for human diarrhoea, and source of infestation is mainly due to consumption of contaminated food. *B. cereus*, a Gram-positive, rod shaped, endospore-forming, motile facultative anaerobic bacteria, can dominate in any given situation, because of its ubiquitous nature and ability to occur in a diversified range of foods^1^. Shortest mean incubation period (0.8 h) and onset of illness within 8 h make *B. cereus* more potent pathogen than the remaining enteric organisms^2^.

There are two types of *B. cereus* food poisoning syndromes caused by two independent toxins. The emetic toxin (<5kDa) is resistant to heat, proteolytic enzyme and low *p*H. This toxin causes nausea and vomiting within 1-5 h after the consumption of contaminated food. The diarrhoeal toxin is a 50kDa heat-labile protein, which is sensitive to proteolytic enzymes and expressed during the late exponential phase of growth. The onset of *B. cereus* mediated infection is about 8-16 h, lasts for 12-24 h, and mostly associated with abdominal pain, profuse watery diarrhoea and tenesmus than nausea and vomiting^3^. Two protein complexes from *B. cereus* isolates, haemolysin BL (HBL) and non-haemolytic enterotoxin (NHE) have been characterized. The haemolysin BL consists of a binding component B and two lytic components L_1_ and L _2_ responsible for enterotoxigenicity of *B. cereus*^4^. The B and L (L_1_ and L_2_) components are encoded in the genes *hblA, hblD* and *hblC*, respectively^5^. These three components may be present in a different composition in *B. cereus*, and all the components together are necessary for the expression haemolysis to occur^6^. Non-haemolytic enterotoxin also consists of three different proteins, A, B and C with the corresponding encoding genes *nheA, nheB* and *nheC*, respectively^7^.

Systematic surveillance and molecular characterization of *B. cereus* isolated from acute diarrhoeal patients were not done in India. Hence, the present study was undertaken to determine the presence of *B. cereus* among patients with acute diarrhoea attending hospital and to characterize the isolates to understand the virulence features as well as clonal nature.

## Material & Methods

### 

#### Collection and processing of stool specimens:

Consecutive diarrhoeal stool specimens collected between October 2006 and September 2008 from outpatients attending the B.C. Roy Hospital and acute diarrhoeal patients admitted and enrolled in the active surveillance at the Infectious Disease Hospital, Kolkata, were included in the present study. Initial enrichment was made in Tryptone soy broth (TSB) (Hi-Media, Mumbai, India) supplemented with 500U/ml of polymyxin B at 37°C for 24-48 h followed by streaking on *B. cereus* selective agar (BCSA) (Hi-Media)^8^. The presumptive identification of *B. cereus* was made on the basis of colony characteristics (peacock blue coloured colonies with a surrounding zone of egg yolk precipitation). The other enteric bacterial pathogens were screened following standard protocols^9^. Diarrhoeagenic *Escherichia coli* were detected by multiplex PCR^10^.

#### Identification of B. cereus:

The presumptive cultures of *B. cereus* obtained from stool specimens were further tested for their motility, endospore formation, followed by species confirmation using biochemical tests with API-50 CHB kit (bioMerieux, La Balme Les Grottes, France).

#### Molecular characterization

Detection of virulence genes - PCR assay was made for the identification of haemolytic BL complex genes namely, *hblC, hblD* and *hblA* and non-haemolytic genes such as *nheA, nheB* and *nheC*. Overnight cultures of *B. cereus* from Luria Bertani (LB) agar (Difco, Detroit, MI, USA) were suspended in 200 µl of sterile distilled water and lysed by boiling for 10 min in a water bath and snap chilled on ice followed by centrifugation at 15000 × g for 5 min. The DNA containing supernatant was transferred into a new microcentrifuge tube and used immediately for PCR. Primers for the detection of the genes of the HBL and NHE complexes are shown in Table I. DNA extract (2.5 µl) was amplified with 0.6 U of Taq polymerase (GeNei, Bangalore, India) using published protocol^11^. PCR products were analyzed by 1.5 per cent agarose gel electrophoresis using 100 bp DNA ladder (NEB, Hitchin, Herts, UK) as molecular weight marker.

**Table I:** List of primers used in the simplex PCR for the detection of virulence associated genes in *B. cereus*

Gene	Primer (5’ →3’)	Amplicon (bp)
*hblA*	B component of haemolysin BL	
	HBLA1:GTGCAGATGTTGATGCCGAT	320
	HBLA2:ATGCCACTGCGTGGACATAT	
*hblD*	L1 component of haemolysin BL	
	LIA:AATCAAGAGCTGTCACGAAT	430
	L1B:CACCAATTGACCATGCTAAT	
*hblC*	L2 component of haemolysin BL	
	L2A:AATGGTCATCGGAACTCTAT	750
	L2B:CTCGCTGTTCTGCTGTTAAT	
*nheA*	A component of non-haemolytic ET	
	nheA 344S: TACGCTAAGGAGGGGCA	500
	nheA 843A: GTTTTTATTGCTTCATCGGCT	
*nheB*	B component of non-haemolytic ET	
	nheB 1500S:CTATCAGCACTTATGGCAG	770
	nheB 2269A:ACTCCTAGCGGTGTTCC	
*nheC*	C component of non-haemolytic ET	
	nheC 2820S:CGGTAGTGATTTGCTGGG	582
	nheC 3401A:CAGCATTCGTACTTGCCAA	

Clonal analysis by pulsed-field gel electrophoresis (PFGE) - The genomic DNA of *B. cereus* was prepared using the protocol of Liu *et al*^12^ with modification. Since, the cell wall of *B. cereus* was difficult to lyse, an early log phase cultures were used to avoid sporulation. In brief, bacterial cells were grown in TSB for 3 h followed by centrifugation with Saline EDTA (SE) buffer (75 mM NaCl, 25 mM EDTA; *p*H 7.5) at 3000 *g*. The cell pellet was suspended in 0.5 ml SE buffer containing 1.5 mg lysozyme (Sigma, St. Louis, MO, USA) and 15 U lysostaphin (Sigma). This suspension was mixed with 0.5 ml 1.5 per cent low melting agarose and dispensed in plug molds. After the lysis and proteinase K treatment, bacterial cells embedded in agarose plugs were washed once for 1 h at room temperature in Tris-EDTA (TE) buffer [10 mM Tris-HCL (*p*H 7.5), 10 mM EDTA], once for 1 h at 37°C in TE buffer containing 1 mM phynylmethylsulphonylfluoride (Sigma) and thrice for 1 h each in TE buffer at 37°C. A slice from each plug (2.5 mm) was cut and incubated overnight with 20 U each of *Sma*I and *Not*I restriction endonuclease (NEB) with the appropriate buffers and the reaction conditions recommended by the manufacturer. After digestion, the plugs were loaded into 1 per cent PFGE agarose (BioRad, Hercules CA, USA) in 0.5 × TBE buffer (Tris, borate, EDTA). Electrophoresis was done in CHEF-Mapper system (BioRad, Hercules CA, USA) for 20 h at 14°C, with an electric field of 6 V/cm, and the pulse time increased from 5.3 to 34.9 sec for *Sma*I and 5 to 80 sec for *Not*I^13^. A bacteriophage lambda ladder (NEB) was used as the molecular weight marker. The reproducibility of the fingerprints was examined by repeated tests.

#### Enterotoxin assay

Haemolysin assay - Culture supernatant of individual isolates of *B. cereus* obtained from overnight culture grown in brain heart infusion broth (Difco, USA) with 0.1 per cent glucose (BHIG) at 37°C at 191 × g assessed for haemolytic activity by agar well plate assay using 5 per cent sheep blood agar (SBA) (bioMeriux, France). Twenty five microlitres of cell-free culture supernatants were added in 5 mm diameter well in the SBA plates and incubated at 30°C and monitored for haemolytic pattern^14^. The ATCC9139 *B. cereus* strain was used as positive control and an *E. coli* K12 used as negative control.

Enterotoxin assay (diarrhoeal type) -For the extraction of *B. cereus* enterotoxin, isolates from pure culture grown on BCSA plates were used after inoculation in 10 ml BHIG and incubation at 37°C for 18 h on a shaker (150 rpm). Culture supernatants obtained by centrifugation at 900 × g for 20 min at 4°C were used immediately for toxin assay using BCET-RPLA (reversed passive latex agglutination) kit (Oxoid, Hampshire, UK) as directed in the manual.

#### Tissue culture assay:

Free bacterial cells of *B. cereus* were harvested after 4 and 18 h growth and washed three times by centrifugation at 402 × g for 10 min each time with Dulbecco’s modified Eagles medium (DMEM, Gibco, Grand Island, NY, USA) and used immediately for HEp-2 monolayers grown in 5 per cent CO_2_ atmosphere at 37°C in DMEM supplemented with 10 per cent foetal calf serum (FCS) in 24-well tissue culture plates in triplicate using published protocol^15^. *Shigella flexneri 2a* and *E. coli* DH5α were used as positive and negative control strains, respectively for invasion assay in the HEp-2 cells.

#### Susceptibility to antimicrobials:

Antimicrobial susceptibility was determined by the disc agar diffusion method^16^. Single colonies of 24 h-old cultures were transferred to 5 ml of TSB (Difco) and incubated at 37°C for 6-8 h. A sterile cotton swab dipped into the TSB growth was applied evenly onto pre-dried Mueller-Hinton agar (Difco) plate. After drying for 15 min, the antimicrobial test discs (HiMedia) were placed aseptically and the plates were incubated at 37°C for 14-19 h. The zones were measured and sensitive, intermediate and resistance was categorized according to the standard methods^16^. ATCC *E. coli* strain 25922 was used as a quality control.

## Results

### 

#### Isolation and identification of B. cereus:

This study was conducted using 1536 stool specimens consecutively collected for two years (October 2006 through September 2008) from B. C. Roy Hospital and Infectious Disease Hospital in Kolkata. Fifty four stool specimens (3.5%) were found to be positive for *B. cereus*. Only a single *B. cereus* isolate was collected from each positive sample and all were confirmed using motility test, endospore formation and biochemical tests with API-50 CHB kit. In the Month-wise detection of *B. cereus* spreading over successive two years, higher incidence of *B. cereus* positive cases was recorded during October, December 2006, March, October and November 2007 (Table II).

**Table II:** Per cent positivity of *B. cereus* isolated from acute diarrhoeal patients during 2006-2008

Month & year	No. of sample	No. of positive sample (%)
Oct 2006	66	6 (9.1)
Nov 2006	99	5 (5)
Dec 2006	39	5 (12.8)
Jan 2007	61	3 (4.9)
Feb 2007	51	0
Mar 2007	58	4 (6.9)
Apr 2007	70	3 (4.3)
May 2007	79	3 (3.8)
Jun 2007	80	4 (5)
Jul 2007	65	0
Aug 2007	53	0
Sep 2007	47	0
Oct 2007	53	4 (7.5)
Nov 2007	90	6 (6.7)
Dec 2007	60	1 (1.7)
Jan 2008	74	2 (2.7)
Feb 2008	56	1 (1.8)
Mar 2008	53	2 (3.8)
Apr 2008	59	1 (1.7)
May 2008	66	2 (3)
Jun 2008	51	1 (2)
Jul 2008	80	1 (1.3)
Aug 2008	68	0
Sep 2008	58	0

*B. cereus* was identified more often in male patients (54%) than females (46%). When age-wise distribution of confirmed *B. cereus* related acute diarrhoeal patients was considered, there was no association between different age groups and only 0.8 per cent of the affected individuals were above 60 yr of age (Table III).

**Table III:** Prevalence of *B. cereus* in different age groups of diarrhoeal patients

	Age groups (yr)				
	<15	15 - <30	30 - <45	45 - >60	>60
No. of samples	599	338	286	192	121
No. of positive samples (%)	23 (3.8)	13 (3.8)	12 (4.2)	5 (2.6)	1 (0.8)

Of the 54 isolates, 42 (78%) were found as sole pathogen associated with diarrhoea. The mixed infections were checked for *Vibrio cholerae, V. parahaemolyticus*, diarrhoeagenic *E. coli, Salmonella* and *Shigella*. Polymicrobial aetiology was detected in the remaining 12 (22%) patients.

#### Detection of virulence genes:

**Fig. 1:**
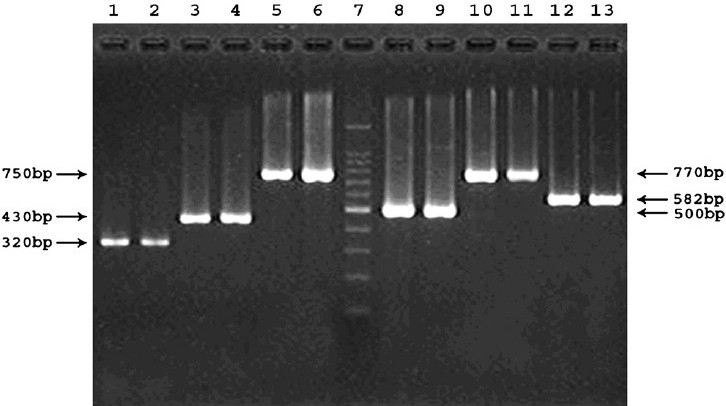
Representative PCR products showing amplicons of six virulence genes in *B. cereus* isolates. Lanes 1 to 2, *hblA* positive isolates M20833 and M20890; 3 to 4, *hblD* positive isolates M20833 and IDH00060; 5 to 6, *hblC* positive isolates M20890 and M21325; 7, molecular size marker 100 bp ladder; 8 to 9, *nheA* positive isolates M20833 and IDH00195; 10 to 11, *nheB* positive isolates IDH00278 and IDH00339; 12 to 13, *nheC* positive isolates M20890 and M21325.

Representative amplicons of *hblA, hblC* and *hblD* encoding the enterotoxin HBL complex and *nheA, nheB* and *nheC* encoding the nonhaemolytic enterotoxin of NHE complex, are shown in Fig. 1. The three genes, *hblA, hblC* and *hblD* were detected in 28 isolates (51.9%). Eight (14.8%) isolates possessed two of the three *hbl* genes and three (5.6%) had only one gene coding the HBL complex. Fifteen (27.8%) *B. cereus* isolates had none of HBL complex. All the three genes *nheA, nheB* and *nheC*, were detected in 48 (88.9%) of 54 *B. cereus* isolates. Five (9.3%) isolates harboured two nhe genes, whereas only one (1.9%) isolate lacked all three genes of NHE complex (Table IV). The nonhaemolytic enterotoxin (NHE) genes *nheA, nheB*, and *nheC* (98, 96 and 91%, respectively) were frequently detected than haemolytic enterotoxin (HBL) genes, *hblA, hblC* and *hblD* (65, 59 and 67%, respectively).

**Table IV:** Results of PCR, haemolysin assay and enterotoxin production of *B. cereus*

Isolate No	*hblA*	*hblD*	*hblC*	*nheA*	*nheB*	*nheC*	Haemolysis	BCET (ng/ml)
VTE2563	+	+	+	+	+	+	+	16
L19524	-	-	-	+	+	+	-	-
L19632	+	+	+	+	+	+	+	128
L20144	-	-	-	+	+	+	-	-
L20166	-	-	-	+	+	+	-	-
L20190	-	-	-	+	+	+	-	-
L21161	+	+	+	+	+	+	+	128
L21500	+	+	+	+	+	+	+	128
L21519	+	+	+	+	+	+	+	64
VTE2582	+	+	+	+	+	+	+	32
VTE2584	+	+	+	+	+	+	+	16
L22622	-	+	+	+	+	+	+	32
VTE2591	-	-	-	+	+	+	+	-
L22959	-	-	-	+	+	+	-	-
VTE2593	+	+	+	+	+	+	+	128
L23188	-	-	-	+	+	+	+	-
M78	+	+	+	+	+	+	+	64
M103	-	-	-	+	+	+	-	-
M293	+	+	+	+	+	+	+	16
M3102	+	+	+	+	+	+	+	8
M3139	-	-	-	+	+	+	-	-
M3551	+	+	+	+	+	+	+	8
VTE2613	+	+	+	+	+	+	+	32
VTE2620	+	+	+	+	+	+	+	16
M4698	+	+	+	+	+	+	+	32
M5007	+	+	+	+	+	+	+	128
M7351	+	+	+	+	+	+	+	64
M7826	+	+	+	+	+	+	+	16
M7985	+	+	+	+	-	+	+	32
M8263	+	+	+	+	+	+	+	128
M8278	+	+	+	+	+	+	+	64
M8432	+	+	+	+	+	+	+	16
VTE2665	-	-	-	+	+	-	-	-
M20833	+	+	+	+	+	+	+	≥256
M20885	+	-	+	+	+	+	+	≥256
M20890	+	-	+	+	+	+	+	128
M21325	-	-	+	+	+	+	+	≥256
M21518	+	-	-	+	+	-	+	-
M21558	+	-	-	+	+	-	+	-
VTE2742	-	+	+	+	+	+	+	≥256
IDH48	-	-	-	+	+	+	-	-
IDH60	+	+	-	+	+	+	+	-
IDH80	+	-	+	+	+	+	+	≥256
IDH106	-	-	-	+	+	-	-	-
IDH195	+	-	+	+	+	+	+	>256
IDH232	-	-	-	-	-	-	-	-
IDH278	-	+	+	+	+	+	+	64
IDH339	-	-	-	+	+	+	-	-
IDH348	+	+	+	+	+	+	+	128
IDH413	+	+	+	+	+	+	+	64
IDH487	+	+	+	+	+	+	+	4
IDH514	+	+	+	+	+	+	+	128
IDH547	-	-	-	+	+	+	-	-
IDH626	+	+	+	+	+	+	+	16

BCET, *B.cereus* enterotoxin

#### Clonal analysis by PFGE:

**Fig. 2:**
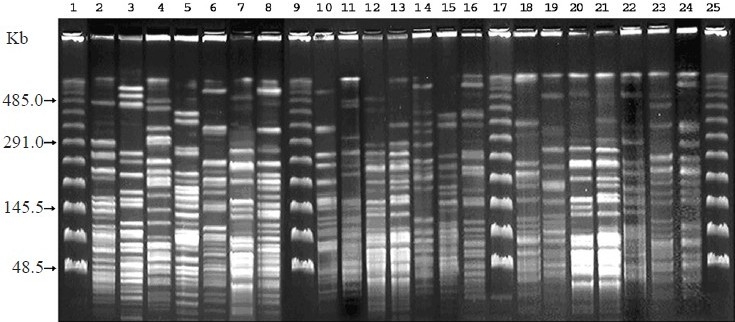
PFGE of *Sma*I digested genomic DNA of *B. cereus* isolates. Lanes 1, 9, 17, and 25 are bacteriophage lamda molecular size makers; 2, ATCC 9139; 3, L20166; 4, L21519; 5, M3139; 6, M8263; 7, M20890; 8, VTE2742; 10, M7985; 11, VTE2665; 12, M21325; 13, M21558; 14, IDH80; 15, IDH232; 16, L20190; 18, M78; 19, M103; 20, M20885; 21, M21518; 22, IDH106; 23, IDH195; 24, VTE2620.

**Fig. 3:**
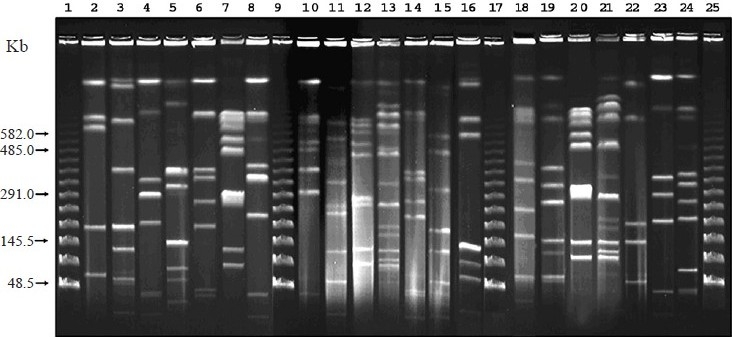
PFGE of *Not* I digested genomic DNA of *B. cereus* isolates. Lanes 1, 9, 17, and 25 are bacteriophage lamda molecular size makers; 2, ATCC 9139; 3, L20166; 4, L21519; 5, M3139; 6, M8263; 7, M20890; 8, VTE2742; 10, M7985; 11, VTE2665; 12, M21325; 13, M21558; 14, IDH80; 15, IDH232; 16, L20190; 18, M78; 19, M103; 20, M20885; 21, M21518; 22, IDH106; 23, IDH195; 24, VTE2620.

Twenty isolates were selected for PFGE analysis based on the PCR results, which represents different combination of *hbl* and *nhe* genes. The fingerprints generated by macrorestriction with *Sma*I comprised about 20-25 bands of approximately 5-500 Kb (Fig. 2), whereas with *Not*I approximately 5-13 bands of approximately 50-700 Kb found (Fig. 3). In the present study, PFGE banding patterns in two isolates were identical (M20885 and M20890) with both the enzymes tested. These isolates also exhibited identical virulence gene profiles.

#### Haemolysin assay:

Majority (76%) of the *B. cereus* isolates exhibited haemolysis (Table IV). Except for isolates VTE2591 and L23188 expression of haemolysin was associated with presence of any of the *hbl* genes. Haemolytic activity was not detected in 13 isolates, which did not harbour any *hbl* genes (Table IV). Discontinuous haemolysis was noticed with *B. cereus* isolates harbouring *hbl*CDA operon, whereas continuous haemolysis was recorded in isolates that lack any one of these genes.

#### Enterotoxin assay:

Qualitative tests on *B. cereus* enterotoxin production using BCET-RPLA kit of 54 isolates showed that 36 (67%) isolates were able to produce BCET on BHIG in a varied concentration ranging from 8 to >256 ng/ml (Table IV). Of these enterotoxigenic isolates, 3 per cent produced 4 ng/ml, 6 per cent produced 8 ng/ml, 19 per cent produced 16 ng/ml, 14 per cent produced 32 ng/ml, 16.5 per cent produced 64 ng/ml, 24 per cent produced 128 ng/ml and 16.5 per cent produced ≥256 ng/ml. There appears to be a association between presence of *hbl* genes and expression of BCET. Of the 18 isolates that were negative in the BCET, 15 did not harbour any *hbl* genes and in 3 *hblC* was absent. There was no association between combination of *hbl* genes and an activity of expressed BCET.

#### Invasion assay:

Based on the PCR results, which represents different combination of *hbl* and *nhe* genes, 20 isolates (same as in PFGE) were also included for invasion assay using HEp-2 cell line. Our result showed that none of the tested isolates invaded into the HEp-2 cells.

#### Susceptibility to antimicrobials:

The susceptibility of 54 *B. cereus* isolates was tested for 10 different antibiotics. All the isolates were susceptible for amikacin, ciprofloxain, gentamicin, and imipenem. Majority of the isolates were also susceptible for ofloxacin and azithromycin. a0 moxyclav and cephalosporins resistance was seen in most of the isolates (Table V).

**Table V:** Antimicrobial resistance of *B. cereus*

Antibiotics (µg/disc)	Zone of inhibition* (mm) R - S	% (n=54)		
		Sensitive (S)	Intermediate (I)	Resistant (R)
Amikacin (30)	≤14-≥17	100	0	0
Amoxyclav (30)	≤19-≥20	0	0	100
Azithromycin (15)	≤13-≥18	80	20	0
Cefexime (5)	≤16-≥23	0	0	100
Ceftriaxone (30)	≤13-≥21	0	45	55
Ceffotaxim (30)	≤14-≥23	5	42	53
Ciprofloxacin (5)	≤15-≥21	100	0	0
Gentamicin (10)	≤12-≥15	100	0	0
Imipenem (10)	≤13-≥16	100	0	0
Ofloxacin (5)	≤12-≥16	95	5	0

* Resistance/susceptible range (Hi-Media, Mumbai, India)

## Discussion

*B. cereus* associated food poisoning is underreported as the types of illnesses are relatively mild and usually last for less than 24 h. Nevertheless, occasional reports of more severe form of diarrhoeal type of illnesses, ubiquitous presence and heat-stable endospore forming nature of the organism underscore the significance of the organism. The unique properties such as heat resistance, endospore forming ability, toxin production and psychrotrophic nature give ample scope for this organism to be a prime cause of public health hazard^17^.

Identification of *B. cereus* was almost constant in all age groups. Contaminated food and warm weather seems to support incidence of *B. cereus*^18^–^21^. In 78 per cent of the patients, we identified the *B. cereus* as a sole pathogen with all the maker virulence genes included in this study thereby indicating their role in causing the disease. However, in 22 per cent of the diarrhoeal patients, polymicrobial aetiology was detected; hence it is difficult to conclude the role of *B. cereus* in these cases. Since, complete data on viral aetiology are not available, we cannot rule out the possibility of mixed infection caused by enteric viruses. The importance of *B. cereus* mediated diarrhoea should be assessed through community based case and control studies.

Two different enterotoxic protein complexes have been characterized, *nhe* genes found in most of the strains in a higher proportion than *hbl* genes. Distribution of these genes in *B. cereus* were more in this study compared to other reports^22^. Our results confirmed previous speculation that in *B. cereus* two or more enterotoxins might be involved in causing diarrhoea in humans^23^. Hansen and Hendriksen^11^ reported that polymorphism among the genes is the likely explanation of the inability to detect all genes in some *B. cereus* isolates by PCR.

The *Not*I PFGE profile has low discriminatory power as it generates less number of DNA bands^12^. The same trend was observed with the clinical isolates of *B. cereus*. On the other hand, Zhong *et al*^24^ found only two to three fragments in the case of *Sma*I digestion, whereas we found 20-25 fragments. Overall, our study demonstrated that *B. cereus* isolated from diarrhoeal patients was not clonal in this region though the virulence gene profiles and expression of BCET was similar. M20885 and M20890 were isolated as a sole pathogen from two patients who were admitted to the IDH on the same day. Both the patients had identical clinical symptoms such as acute watery diarrhoea and dehydration. However, demographic information showed that they were not related. Since these two isolates were clonally identical as evidenced from the PFGE, these would have infected from a common source.

The determination of haemolytic activity on SBA led to interesting results. All the isolates that were positive for *hbl* genes in PCR exhibited haemolysis in SBA. Two isolates of *B. cereus* found weakly positive for haemolysis, but failed to show PCR amplification for *hbl* genes. This could be due to sequence variation in the binding site of the primers^25^. While studying the distribution of *hblCDA* and *nheABC* genes, Ngamwongsatit *et al*^26^ have shown that detection of these genes were increased when alternative primers were used, with high specificity of the assay. Similar to the finding of Thaenthanee *et al*^27^, we have detected higher number (51.8%) of *B. cereus* isolates that displayed discontinuous haemolysis.

To define the extent of toxin production by *B. cereus*, an analysis of BCET was made. The BCET-RPLA test does not specifically react with diarrhoeal toxin, but with the L_2_ component of HBL complex. Since, the non-enterotoxigenic isolates lack *hbl* gene specially the L_2_ component, the BCET-RPLA kit is useful in the identification of this virulent factor^28^. Expression of BCET was detected in more number of isolates in this study than from other reports^23^.

Our result showed that none of the tested isolates invaded into the HEp-2 cells. Rowan *et al*^15^ found 91 per cent *Bacillus* species, of which 100 per cent *B. cereus* isolates were unable to invade HEp-2 cells, whereas Rowan *et al*^29^ showed 49 per cent of different *Bacillus* species showed various levels of invasion on HEp-2 cells of which 75 per cent of *B. cereus* strains were non-invasive to HEp-2 cells.

Antimicrobial susceptibility testing guides for the empirical use of antibiotics and proper management of diarrhoea. In this study, majority of *B. cereus* isolates were resistant to amoxyclav and cephalosporins. This trend seems common in other regions as well^30^. *B. cereus* isolated from different sources was generally resistant to penicillin, ampicillin, cephalosporins, and trimehoprim and susceptible for clindamycin, erythromycin, chloramphenicol, vancomycin, imipenem, aminoglycosides and ciprofloxacin^31^.

In conclusion, our results showed the importance of *B. cereus* among hospitalized patients with acute diarrhoea in Kolkata. PCR amplification of toxin genes and PFGE analysis showed diverse virulence factors as well as of clonality of the isolates from acute diarrhoeal patients. Knowledge of spectrum of antibiotic susceptibility will possibly become a guide to empirical therapy to shorten the morbidity in acute stage, as microbial quality assay from stool specimens is not a routine practice in this part of the world. Community based studies are needed to know the diarrhoeal disease burden due to this pathogen in this region.
